# Anthropometry of World-Class Elite Handball Players According to the Playing Position: Reports From Men’s Handball World Championship 2013

**DOI:** 10.2478/hukin-2013-0084

**Published:** 2013-12-31

**Authors:** Hamid Ghobadi, Hamid Rajabi, Babak Farzad, Mahdi Bayati, Ian Jeffreys

**Affiliations:** 1 Department of Exercise Physiology, Faculty of Physical Education & Sports Science, Kharazmi University, Tehran, Iran.; 2 Department of Exercise Physiology, Faculty of Physical Education & Sports Science, Kharazmi University, Tehran, Iran.; 3 Department of Exercise Physiology, Faculty of Physical Education & Sports Science, Kharazmi University, Tehran, Iran.; 4 Department of Physical Education and Sports Science, Faculty of Humanities, Tarbiat Modares University, Tehran, Iran.; 5 Department of Science and Sport, University of South Wales, Pontypridd, Wales, United Kingdom.

**Keywords:** Body Mass, Handball, Standing Stature, Body Mass Index, Talent Identification

## Abstract

Identifying the anthropometric measures of successful and less successful handball players may be helpful in developing a talent identification and development model, allowing for the determination of key physical capacities required for elite performance. The purpose of the study was to describe the anthropometric characteristics, including age, standing stature, body mass and body mass index (BMI) in handball players who participated in the 2013 Men’s Handball World Championships. Secondly, the objective was to identify the possible differences in these parameters in terms of individual playing positions (goalkeeper, back, center back, wing, line player). Rosters with handball player’s age, standing stature, and body mass were obtained from the International Handball Federation website. The research material included 409 handball players (24 teams). National teams were organized by their ranks and sub-grouped using their continents and playing positions. The results of the analyses of variance demonstrated significant differences in age (F=2.30; p=0.044; Partial ŋ2=0.028), standing stature (F=14.02; p=0.0001; Partial ŋ2=0.148), and body mass (F=5.88; p=0.0001; Partial ŋ2=0.068) among the groups (G1–G6). Players in G1 had the highest standing stature and body mass, while players in G6 had the lowest age and body mass values. The backs and line players were the tallest. In addition, the measurement of body mass showed that the line players had the highest body mass and BMI values. In conclusion, this study presented anthropometric data that differentiated levels of success in male handball teams playing in the 2013 world championships. This information should serve as a reference for the average standing stature, body mass, and BMI of handball players for particular positions at the professional level.

## Introduction

Handball is an Olympic team sport which has been played since the 1972 Games in Munich with further major international competitions encompassing world championships, continental championships, and international tournaments, as well as major club championships being played throughout the world. From the point of view of physical performance, handball is a complex, intermittent sport game, played over 60 minutes, which requires efforts of maximum intensity in a short period of time, where players jump, run, and throw the ball at a high velocity, followed by low intensity recovery periods. Clearly, given the physical challenges of the game it requires significant physical preparation in order to compete successfully (Ziv and Lidor, 2009; Moncef et al., 2012). Handball performance is, to some extent, affected by the anthropometric characteristics of athletes and it is possible that such characteristics differentiate players of a different competitive level (Chaouachi et al., 2009; Milanese et al., 2011). It has been proposed that each specific position in handball requires unique physiological and physical attributes relating to the technical and tactical requirements of each position in order to maximize performance on the playing field (Vila et al., 2012; Chaouachi et al., 2009). Data regarding the anthropometric characteristics of elite handball players provides specific information that may assist in directing the players to the most appropriate playing positions (Sibila and Pori, 2009). In addition, coaches and investigators may be able to utilize this data in the process of talent selection. Talent selection involves the ongoing process of identifying players at various stages who demonstrate prerequisite standards of performance for inclusion in a particular team. It is focused on choosing the most appropriate individual, or group of individuals, who can best carry out the task within a specific context (Bouchard et al., 1997; Mohamed et al., 2009). Recognizing true talents for a particular sport is a very complex process and requires good knowledge of the anthropometric and physiological characteristics that are relevant for top performance in that particular sport (Srhoj et al., 2006). Anthropometric characteristics of elite handball players have been suggested to be a biomarker in determining the athletic potential of an individual (Mohamed et al., 2009; Milanese et al., 2011). In general, more successful teams are taller and have lower body fat than less successful teams (Hasan et al., 2007). Hasan et al. (2007) compared the anthropometric profiles of English handball players with Asian ones from different countries. The results demonstrated significant differences in body height, body mass, percentage of body fat, adiposity and muscle mass among teams. In addition, there was no significant difference in age, body height, body mass, percentage of body fat, and muscle mass based on the positions (Hasan et al., 2007).

Thus, the main aim of this study was to describe the anthropometric profile of elite handball players who participated in the world championships from five different continents and compare these characteristics based on different playing positions (goalkeeper, back, center back, line player, and wing).

## Material and Methods

The anthropometric characteristics of the handball players who participated in the 2013 World Men’s Handball Championship were analyzed. Rosters with player’s age, standing stature, and body mass were obtained from the International Handball Federation website (www.ihf.info). All team rosters and all players who were registered for the tournament were included. This comprised a total of 24 teams, with 409 handball players. For the purpose of analysis, the age, standing stature, body mass, and playing position of all 409 male handball players from 24 countries and 5 continents were recorded and body mass index (BMI) of the players calculated. National teams were organized by their ranks and sub-grouped using their continents and playing positions.

## Statistical analysis

All results are reported as a mean ± SD. The Kolmogorov-Smirnov test was used to verify the normality of the distribution. In addition, differences of age, body height, body mass and body mass index across groups (G1–G6), playing positions and continents were analyzed with oneway analysis of variance (ANOVA). When a significant difference was revealed, the Dunnett’s T3 or Scheffe’s post hoc test was used to specify where the difference occurred. When the homogeneity of variance was established, the Scheffe’s post hoc test was used; otherwise, we applied the Dunnett’s T3 post hoc test. Effect size was calculated using Partial Eta Squared (Partial ŋ2). Playing position-adjusted partial correlation coefficients were calculated to investigate the association between team rank and age and anthropometric characteristics. The level of significance was set at *p* ≤ 0.05. All data were analyzed by the SPSS software package (SPSS for Windows; SPSS Inc., Chicago, IL, USA; Version 16.00).

## Results

Anthropometric characteristics of male handball players from the 2013 World Championships, grouped according to their ranks, are presented in Table 1. France (Rank 6), Croatia (Rank 3), Spain (Rank 1) and Saudi Arabia (Rank 19) had the highest age, standing stature, body mass and BMI values, respectively. However, Australia (age), Saudi Arabia (standing stature) and South Korea (body mas and BMI) had the lowest values.

The results of the analyses of variance demonstrated significant differences in age (F=2.30; p=0.044; Partial ŋ^2^=0.028), standing stature (F=14.02; p=0.0001; Partial ŋ^2^=0.148), and body mass (F=5.88; p=0.0001; Partial ŋ^2^=0.068) among the groups (Table 2). Players in G1 had the highest standing stature and body mass, while players in G6 had the lowest age and body mass values.

Anthropometric characteristics of male handball players according to their positions are presented in Table 3. The results for the analyses of variance demonstrated significant differences in age (F=4.38; p=0.002; Partial ŋ^2^=0.042), standing stature (F=28.62; p=0.0001; Partial ŋ^2^=0.221), body mass (F=38.46; p=0.0001; Partial ŋ^2^=0.276), and BMI (F=12.57; p=0.0001; Partial ŋ^2^=0.111) among the positions. The backs and line players were taller than other players. In addition, the measurement of body mass showed that the line players had the highest body mass and BMI values (Table 3).

For further analysis of the data, the teams were divided into five groups according to their geographical location (Table 4). European players had significantly higher values in age, standing stature and body mass than the others.

Furthermore, position-adjusted partial correlations showed that there were negative associations between the ranks of the teams and age (r = −0.150; p=0.002), standing stature (r = −0.398; p=0.0001), and body mass (r = −0.253; p=0.0001; Table 5).

## Discussion

Previous reports have shown that body stature and morphological characteristics can determine the selection of participants in many sports (Hasan et al., 2007). To succeed in a sport discipline, it is often important to have specific anthropometric attributes (Ziv and Lidor, 2009). Handball involves frequent body contact and several high-intensity actions as part of a match play (Póvoas et al., 2012). Knowledge of the physical characteristics of handball players can provide insight into the individual factors which influence the players’ performance in the game (Hasan et al., 2007). The most striking comparison of anthropometric features of handball players in the present study was the difference in standing stature and body mass among the groups. Players in G1 had the highest standing stature and body mass when compared with players in G6, while BMI was not different across the groups (Table 2). In one study, elite (who were members of the current Spanish handball champions) and amateur handball players of the same age were compared. The elite players were taller, heavier and had a higher fat-free mass and higher BMI than the amateur players (Gorostiaga et al., 2005). Marques and Gonzalez-Badillo (2006) reported the stature and body mass of the high-level team handball players were 184.2 ± 13.1 cm and 84.8 ± 13.1 kg, respectively (Marques and Gonzalez-Badillo, 2006). Marques et al. (2007) also reported the stature and body mass of the senior elite male team-handball players were 182.1 ± 6.7 cm and 82.5 ± 12.2 kg, respectively (Marques et al., 2007). However, the values of both studies were lower than players in G6 of the present study. Lean mass, especially in the upper body, i.e. the body segment most involved in throwing, is the key feature of handball (Milanese et al., 2011). However, it needs to be noted that this trend for heavier and taller players has not been universally found as Hassan et al. (2007) found no significant differences in age, body mass and muscle mass between successful and unsuccessful players. However, the successful players were taller than the unsuccessful players. Negative associations were found between age, body mass, and fat mass with maximal oxygen uptake, and maximal velocity obtained in the Yo-Yo recovery test in 42 male handball players who were members of three elite Tunisian handball teams (Moncef et al., 2012). The more successful teams had higher mean body mass values in our study. This trend towards heavier and taller players should also play a role in an appropriate talent identification and development program. Discriminate analysis between successful and less successful teams revealed that standing stature and body mass are important variables for talent identification, and therefore, should play an important role in developing an effective program of physical development, where the attainment of an appropriate degree of fat free muscle mass should be one of the goals. An interesting finding of the present study was that the mean age of the more successful teams (players in G1 and G2) was higher than in the other groups. In this way, playing experience seems to be an important element in team success. This may also reflect the fact that top performers in European countries may have a longer athletic career enabling them to play for their national teams for a longer time. A further study supports the role of age in successful teams, reporting that elite female players were older than sub-elite by about nine years; this may have been for the obvious reason that more experienced players play in elite settings (Milanese et al., 2011). However, in our sample there was just a significant difference between G2 and G6.

Certain anthropological characteristics have significant influence on the position-related performance in sports (Sibila and Pori, 2009). It would appear that the anthropometric characteristics of elite handball players appear to be different across positions. When anthropometric data from the sample were analyzed according to playing positions, several significant differences were found (Tables 3). Differences in key body parameters such as stature, body mass, and BMI between players in different positions is in line with previously reported data in male and female handballers (Chaouachi et al., 2009; Sibila and Pori, 2009; Srhoj et al., 2002; Milanese et al., 2011). In contrast, Asian players were found to be relatively homogeneous across the different positions (Hasan et al., 2007).Wings showed a tendency to differ from any other position (Table 3), with the greatest difference being with line players: wings were significantly lighter and shorter, which is consistent with previous investigations reporting that wings were significantly lighter and shorter with less lean body mass and fat mass (Milanese et al., 2011; Sibila and Pori, 2009; Srhoj et al., 2002). This may be explained by the roles of wings, who are required to rapidly shuttle from defense to offense and often, throwing at the goal without significant contact with the rival defensive players, attempting to exploit speed and agility (Milanese et al., 2011). Wings were the shortest players, which is consistent with a study on elite Croatian handball players (Sporis et al., 2010). Sporis et al. (2010) examined anthropometric characteristics of ninety-two elite Croatian handball players. They reported that the goalkeepers were the oldest, the wings were the shortest and the pivots were the tallest players in the team, while the backcourt players had a lower percentage of body fat. In the present study, line players (pivots) were heaviest while backcourt and line players were tallest (Table 3). In addition, the goalkeepers were older than the centre backcourt, backcourt and wing players (p<0.05). Chaouachi et al. (2009) also investigated the anthropometric and performance characteristics of twenty-one elite Tunisian national handball players according to their playing positions, and found the only differences observed between the positions were in the height values of backs and wings, and in the percentage body fat of goalkeepers and backs. In addition, they did not find a significant correlation between body height and body mass with standing throw velocity (Chaouachi et al., 2009). In 78 handball players who were members of the Slovenian junior and senior national team, Sibila and Pori (2009) demonstrated that wings were significantly different from the other player groups in terms of their morphological body characteristics. The values of wings’ body height and body mass were significantly lower than those of players in the other groups. While the backs and pivots had the highest body mass and backs had highest standing stature (Sibila and Pori, 2009).

When players were grouped according to their geographic location, interesting differences between particular continents could have been found. European players were older, taller and heavier than the others (p<0.05), but BMI did not differ among the continents (Table 4). Hasan et al. (2007) compared the anthropometric profiles of English handball players with Asian ones from different countries. The results demonstrated significant differences in body height, body mass, percentage of body fat, adiposity and muscle mass among teams. The mean age of the English players was significantly lower than the East Asia and West Asia handball players. Also, the East Asian players were taller than the West Asian and English players, and West Asian players were taller than the English ones. However, their body mass and muscle mass were not significantly different (Hasan et al., 2007). It must be kept in mind though that English handball players do not represent elite performance within a world and European context, and this can explain the differences found in this study where European elite teams were analyzed. In our sample, Asian and Oceania players had the lowest standing stature and body mass, respectively.

Pearson correlations or multiple regressions are often used to identify which anthropometric characteristics or physical capabilities can predict athletic performance (Chaouachi et al., 2009). Strength, power, and throwing velocity are important factors in elite handball players (Gorostiaga et al., 2006) and it has been suggested that this can be affected by body mass and stature (Chaouachi et al., 2009). Position-adjusted partial correlations showed that there were negative associations between the ranks of the teams and age (r = −0.150; p=0.002), standing stature (r = −0.398; p=0.0001), and body mass (r = −0.253; p=0.0001; Table 5). Chaouachi et al. (2009) demonstrated that body height and body mass were not significantly related to standing throw velocity in Tunisian national handball players (Chaouachi et al., 2009).

In conclusion, this study presented anthropometric data on more and less successful male handball teams that participated in the 2013 World Championships. Furthermore, this study confirms and expands on previous data about anthropometric differences among playing positions and continents in handball. The measurement of anthropometric characteristics provides an insight into the current status of handball players, allowing coaches to evaluate typical characteristics of elite performers. This information should serve as a reference for what the average standing stature, body mass, and BMI of handball players may be for positions at the professional level. This date can be used to develop a model of elite handball performance which can be used to supplement talent identification programs, and also in the construction of effective player development programs.

## Figures and Tables

**Table 1:** Anthropometric characteristics (mean±SD) of male handball players in the 2013 World Championships grouped according to their ranks

Rank	Country	n	Age (yr)	Standing Stature (cm)	Body Mass (kg)	Body Mass Index (kg/m^2^)
1	Spain	16	28.19±4.05	192.88±7.60	96.88±11.23	26.02±2.44
2	Denmark	17	27.47±4.50	194.00±7.51	94.82±10.37	25.17±2.16
3	Croatia	18	25.89±3.70	194.39±8.99	96.72±12.64	25.49±1.79
4	Slovenia	17	26.88±4.16	191.53±7.08	94.53±9.97	25.73±1.90
5	Germany	16	27.00±3.36	193.44±4.81	92.19±6.37	24.62±1.17
6	France	16	29.44±5.31	191.00±6.54	92.69±9.74	25.35±1.69
7	Russia	16	28.56±3.94	192.12±7.91	96.44±12.79	26.06±2.58
8	Hungry	16	27.88±3.55	193.44±6.15	96.75±9.78	25.83±2.08
9	Poland	18	28.89±3.89	193.94±6.86	93.00±9.62	24.65±1.21
10	Serbia	18	26.28±4.95	191.56±6.73	94.56±9.42	25.72±1.81
11	Tunisia	17	24.76±4.28	190.71±5.58	90.76±8.80	24.96±2.35
12	Iceland	17	27.71±4.46	191.82±5.59	92.47±6.53	25.12±1.37
13	Brazil	16	25.62±3.75	190.19±4.91	95.12±7.63	26.28±1.74
14	Fyro Macedonia	17	28.82±3.62	189.53±4.92	92.65±9.58	25.76±2.11
15	Belarus	17	27.24±4.07	193.41±6.03	95.82±11.01	25.55±1.98
16	Egypt	18	24.78±3.93	184.83±4.12	90.17±11.84	26.34±2.87
17	Algeria	17	27.18±4.37	188.12±3.21	88.65±4.83	25.04±1.22
18	Argentina	18	25.89±3.34	188.56±5.86	90.00±8.17	25.40±3.09
19	Saudi Arabia	18	26.61±4.46	183.72±6.25	90.00±8.67	26.66±2.25
20	Qatar	17	26.76±4.49	185.47±5.83	87.88±11.59	25.47±2.39
21	South Korea	18	26.22±3.79	186.72±4.56	85.22±7.76	24.43±1.93
22	Montenegro	17	28.35±4.84	190.06±5.83	96.18±8.39	26.65±2.45
23	Chile	18	24.78±3.73	186.00±6.04	88.94±6.85	25.69±1.37
24	Australia	16	23.88±3.34	186.19±6.89	85.50±7.79	24.65±1.72
	Total	409	26.86±4.24	190.10±6.82	92.37±9.80	25.53±2.09

**Table 2:** Anthropometric characteristics of male handball players in the 2013 World Championship categorized into six groups

	n	Age (yr)	Standing Stature (cm)	Body Mass (kg)	Body Mass Index (kg/m^2^)
G1	68	27.7±4.10	193.22±7.75	95.74±10.92	25.59±2.05
G2	64	28.22±4.11	192.50±6.38	94.52±9.92	25.46±1.98
G3	70	26.93±4.59	192.03±6.22^‡^	92.73±8.62	25.12±1.74
G4	68	26.60±4.08	189.41±5.83^†§^	93.37±10.23	25.98±2.21
G5	70	26.60±4.12	186.46±5.69^†*^	89.16±8.51^†*^	25.66±2.37
G6	69	25.83±4.23^*^	187.23±5.96^†*^	88.96±8.73^†*^	25.36±2.06

G1: Rank 1 through 4; G2: Rank 5 through 8; G3: Rank 9 through 12; G4: Rank 13 through 16; G5: Rank 17 through 20; G6: Rank 21 through 24.

† Significantly different from G1;

* Significantly different from G2;

§ Significantly different from G5;

‡ Significantly different from G5 and G6. All values are expressed as mean±SD.

**Table 3:** Anthropometric characteristics of male handball players in the 2013 World Championship grouped according to their positions

	n	Age (yr)	Standing Stature (cm)	Body Mass (kg)	Body Mass Index (kg/m^2^)
Goalkeeper	55	28.82±4.82	191.89±5.18	95.60±10.45	25.97±2.80
Back	135	26.40±4.20^*^	192.62±6.65	94.17±8.20	25.37±1.76
Centre Back	55	26.09±3.92^*^	188.05±5.96^*†^	89.42±8.12^*†^	25.28±1.97
Wing	97	26.45±3.80^*^	185.01±5.46^*†‡^	84.66±6.44^*†‡^	24.73±1.49^*†^
Line Player	67	27.39±4.29	192.61±6.33^‡§^	99.66±9.47^†‡§^	26.85±2.18^†‡§^

* Significantly different from Goalkeeper;

† Significantly different from Back;

‡ Significantly different from Centre Back;

§ Significantly different from Wing. All values are expressed as mean±SD.

**Table 4:** Anthropometric characteristics of male handball players in the 2013 World Championship grouped according to their continents

	n	Age (yr)	Standing Stature (cm)	Body Mass (kg)	Body Mass Index (kg/m^2^)
Europe	236	27.74±4.21	192.37±6.69	94.69±9.86	25.55±1.97
Africa	52	25.56±4.26^*^	187.83±4.96^*^	89.87±8.89^*^	25.47±2.31
Asia	53	26.53±4.18	185.30±5.62^*^	87.70±9.47^*^	25.52±2.34
America	52	25.42±3.57^*^	188.17±5.81^*^	91.21±7.88	25.77±2.20
Oceania	16	23.87±3.34^*^	186.19±6.89^*^	85.50±7.79^*^	24.65±1.72

* Significantly different from Europe. All values are expressed as mean±SD.

**Table 5:** Position-adjusted partial correlation coefficient among anthropometric measures and ranks of teams

Age (yr)	−0.150 p=0.002
Standing Stature (cm)	−0.398 p=0.0001
Body Mass (kg)	−0.253 p=0.0001
Body Mass Index (kg/m^2^)	0.021 p=0.669
